# Supplementary material for: The effect of monetary incentive on survey response for vulnerable children and youths: A randomized controlled trial
Source: PLoS One. 2020 May 12;15(5):e0233025. doi: 10.1371/journal.pone.0233025 (PMC7217457; doi:10.1371/journal.pone.0233025)
Supplement: Attachment — Submitted filename: Response to reviewers .docx [file pone.0233025.s002.docx]

Dear Editor

Thank you for the review. Below is the response to the reviewers’ comments.

Kind regards,

Jan Pejtersen

| Comment | Response | Change, Page and line |
| --- | --- | --- |
| 1. Please ensure that your manuscript meets PLOS ONE's style requirements, including those for file naming. | We have changed the names for figure1 and the dataset. | Fig1.tif  S1 Dataset.xlsx |
| 2. Please include the ClinicalTrials.gov Identifier in your manuscript (NCT01741675) | The Identifier NCT01741675 was included. | P3, L29/30 and P10, L197/198  ID number SFI2251-26.11.2012 changed to *Identifier: NCT01741675* |
| 3. Please clarify in your manuscript that your ethics committee specifically waived the need for ethics approval. | This has been clarified in the manuscript. See also (4). | P6, L113/117  The RCT study was submitted to the National Committee on Health Research Ethics (reference number VEK H-1-2012-FDP-79), who declared that questionnaire surveys do not need approval by the ethics committee in Denmark. The notification exemption for studies that only include questionnaire surveys is described in part 4, section 14(2) of the Danish Act on Research Ethics Review of Health Research Projects [19]. |
| 4. You indicated in your ethics statement that you obtained written informed consent from a parent for participants under 16 years old. Please clarify whether parental consent was also obtained from participants aged 16 and 17. If not, please clarify whether the research ethics committee specifically waived the need for their consent. Please add this information to your methods section. | The description of the ethics issue has been elaborated in the method section. For the participants under 16 parental consent was obtained. All other gave consent themselves. The ethics committee only responded that the study was not a medical study but a questionnaire survey and that this type of study does not need ethical approval in Denmark.  We have inserted a reference to the law for the ethics committee explaining about questionnaire surveys, see above (3). | *P6 L109-112*  In the evaluation study, written informed consent was obtained for all participants. Approximately 70 % of the children and youths who participated in the social initiative gave consent to participate in the evaluation study. For participants under 16 years old, written informed consent was obtained from a parent, and for all other participants, consent was given by the participants themselves.. |
| 5. PLOS requires an ORCID iD for the corresponding author in Editorial Manager on papers submitted after December 6th, 2016. Please ensure that you have an ORCID iD and that it is validated in Editorial Manager. To do this, go to ‘Update my Information’ (in the upper left-hand corner of the main menu), and click on the Fetch/Validate link next to the ORCID field. This will take you to the ORCID site and allow you to create a new iD or authenticate a pre-existing iD in Editorial Manager. Please see the following video for instructions on linking an ORCID iD to your Editorial Manager account: https://www.youtube.com/watch?v=_xcclfuvtxQ | I had multiple accounts for PLOS One with only one of them validated in Editorial manager. This has now been solved. | The ORCID ID has been validated in Editorial Manager. |
| 6. Please include captions for your Supporting Information files at the end of your manuscript, and update any in-text citations to match accordingly. Please see our Supporting Information guidelines for more information: http://journals.plos.org/plosone/s/supporting-information. | We have included captions at the end of the manuscript. | P24 L 475-477  Supporting Information  S1 Dataset Dataset for the study  (xlsx) |
| Reviewer #1: |  |  |
| 1. The authors mentioned they used vulnerable children and youths in this research but its not clarified why they are considered as vulnerable groups. | We have elaborated on this in the method section. We have described the established intervention study in more details. | P6, L95-108  Participants were taken from an evaluation study of a social initiative aimed at creating networks for different groups of vulnerable children and youths aged 8-23 years [18]. Both the social initiative and the evaluation study were initiated by the Danish National Board of Social Services and were performed in ten municipalities in Denmark. The main purpose of the social initiative was to strengthen the social skills of the participants as well as their ability to create social relations. The children and youth all came from families with severe social problems. Moreover, they were vulnerable and lonely, because they had an inadequate or severely restricted social network or were excluded from their peer group. Participants came from three main groups: children in foster care or former foster care youths; children from vulnerable families; and lonely children and youths. The children from vulnerable families included children from families with a mentally ill or physically disabled parent or sibling, children of parents with alcohol abuse problems and children raised in violent families. The group characterized as lonely children and youths was a group of children and youths that did not fit into the other two groups. |
| 2. I think it would be good update why non response rate among the children and youth is a major problem. | This is a general problem in surveys. We have elaborated on this in the introduction. | *P3, L39-41*  The bias can occur when the non-responders are systematically different from those who participate in the survey. The likelihood of bias decreases with increasing response rate. |
| 3. In introduction, I feel the reference number 1 was outdated and more recent one could be found and utilized. | We have searched the literature and were not able to find a newer review on the topic than the review by Galea and Tracy. Other studies (also new studies) on survey response rates also refer to the Galea and Tracy study. |  |
| 4. Why children below 8 years old were included. Isn't it obvious they would not know the importance in participating in a research through postal survey. | Children below 8 years old were not included. The established intervention study was aiming at children and youths aged 8-23. However, as the present RCT study was a follow-up study of the established intervention study, the youngest child was 11 years old (L217/218). |  |
| 5. line 126 to 128 " Participants with a number lower than 0.5 were allocated to the control group, participants with a number greater than 0.5 were allocated to the intervention group". This is not clear. | We have reorganized the paragraph by moving a sentence and tried to make it clearer. | P7, L39-42  Participants assigned with a number lower than 0.5 were allocated to the control group, participants assigned with a number greater than 0.5 were allocated to the intervention group. We used the Rand (‘uniform’) function in SAS to create the randomization sequence. |
| 6. Very minimal statistical analysis performed. Table 1 and Table 5 can be merged and findings can be presented together. | We have merged table 1 and table 5 together as suggested. |  |
| I am still not clear how this research will contribute to the existing body of knowledge. | In our literature search in the introduction, we did not find any studies that have looked at how to increase response rates for vulnerable children and youths that come from families with severe **social** problems. |  |
| Reviewer #2: Note: I didn't see a link to the full data set within the PDF file I was provided. However, maybe the authors submitted this separately? i responded with a "no" to the question above (#3) as I don't see such a link, but it may exist outside of this PDF. | The full dataset was submitted in an excel file. This has been clarified under the heading  Supporting information | P24 L 475-477  Supporting Information  S1 Dataset Dataset for the study  (xlsx) |
| Note: I answered "I don't know" to question 2 as it was unclear from the manuscript how the randomization was completed. It is possible it was done correctly, but I don't know. The authors need to provide more information within the methods section of their paper. | We have tried to make it clearer. We have reorganized the description of the randomization. | P8, L142-147  Within each wave of the study, participants were sorted according to their identification number and were assigned a random number between 0 and 1. Participants assigned with a number lower than 0.5 were allocated to the control group; participants assigned with a number greater than 0.5 were allocated to the intervention group. We used the Rand (‘uniform’) function in SAS to create the randomization sequence. |
| Overall, the authors seem to be filling an important gap in the literature (conducting on RCT on the effectiveness of monetary incentives on an under-studied population). They also measure these effects on survey responses themselves. The use of an RCT is a reasonable way to meet their research objectives. However, there are a number of serious issues with the manuscript, including the extent to which these findings can be generalized to other populations, methodological inconsistencies, a lack of clarity regarding the make-up of the study sample, as well as various grammatical issues. | Thank you for recognizing the need for studies involving this population. We have tried to response to your points below. |  |
| GRAMMAR  This paper could benefit tremendously from professional editing with attention to both grammar and diction. The language could also be more formal, and the conclusion in particular could be more fleshed out (it is only 2 sentences). Noting just a few issues on the first page or two: | The paper has already been edited by a professional editing bureau. However, the paper has now been through a second quality check by the editing bureau.  The conclusion is more detailed described in the beginning of the discussion. Therefore, the text under the heading ‘conclusion’ was meant to be a short and precise description of the conclusion. |  |
| (1) Noun/ verb disagreements: Different types of incentive(S); age group(S). | We have corrected this. |  |
| (2) The sentence starting on line 47 “Different types of incentive” is repetitive with the second half of that sentence. | We do not quite understand this comment. However, we have tried to change the sentence. | P4, L49-52  Different types of incentives were examined in most of the trials, and it was seen that not only did monetary incentives significantly increase the response rate to postal questionnaires (odds ratio (OR) 1.87), they also had a greater effect than non-monetary incentives (OR 1.62). |
| (3) The sentence “The participating children and youths were vulnerable and lonely; because of they had inadequate or severely restricted social network or were excluded from their peer group” has multiple issues. | We have changed the description of the children and youths. | P6, L100-103  The children and youth all came from families with severe social problems. Moreover, they were vulnerable and lonely, because they had an inadequate or severely restricted social network or were excluded from their peer group. |
| STUDY LOCATION  It would be useful to have a better sense of the location of the study in order to understand to what degree we can make generalizations about this study. Does this study take place in Denmark? Where precisely? Can we find out more about the population? | We have described the study location, the established intervention study and the population in more details.  We have described the 3 studies involved.  The social initiative in ten municipalities. The evaluation of the social initiative. The present RCT study is a follow-up study as part of the evaluation study (al-ready described in the manuscript).  We have included more details on the study population in table 1. | P 6, L 95-101  Participants were taken from an evaluation study of a social initiative aimed at creating networks for different groups of vulnerable children and youths aged 8-23 years [18]. Both the social initiative and the evaluation study were initiated by the Danish National Board of Social Services and were performed in ten municipalities in Denmark. The main purpose of the social initiative was to strengthen the social skills of the participants as well as their ability to create social relations. The children and youth all came from families with severe social problems. |
| SAMPLE  The authors state that the present RCT is limited to those that gave consent. What percentage of participants in this program gave consent to participate? How representative is this group of the target population? | We do not have information on the participants who did not give consent to participate in the evaluation of the program. However around 70 % gave consent to participate in the evaluation of the program. | P6, L109-111  In the evaluation study, written informed consent was obtained for all participants. Approximately 70 % of the children and youths who participated in the social initiative gave consent to participate in the evaluation study. |
| The authors state that there are three groups of participants: “lonely” children, children in foster care, and vulnerable children. How is this determination made? How were participants identified generally? - particularly for the “lonely children”? What does this mean practically? In what kind of family situation are these children living? Again, this information would provide context and offer information regarding the external validity of this study. | We have elaborated on the description of the established study in general and also specific on the population, see STUDY LOCATION. We have corrected the language.  We have rewritten the description of the groups of children and youths. | P6, L103-108  Participants came from three main groups: children in foster care or former foster care youths; children from vulnerable families; and lonely children and youths. The children from vulnerable families included children from families with a mentally ill or physically disabled parent or sibling, children of parents with alcohol abuse problems and children raised in violent families. The group characterized as lonely children and youths was a group of children and youths that did not fit into the other two groups. |
| INTERVENTION  In terms of the RCT itself, how do we know that the subjects under study themselves responded to this incentive? (And not the parents or caregivers of the children etc.)? - Especially for younger participants (8-12 or so). Also, how long was the questionnaire/ roughly how long did it take? This information could be provided on lines 144-155. | To know how the incentive worked in details is off course interesting, but this was beyond the scope of the present study. We just wanted to study if the incentive had an effect on the response rate for this group of subjects.  The length of the questionnaire (90-91 items) is described in L165-169. |  |
| METHODS  The authors state that they randomized the treatment using various procedures in SAS, but the treatment and control groups are quite different in size (146 versus 121). Why is this? Was the randomization successful? Further, in Table 1, it is customary to also provide statistical tests of means post-randomization to verify that the randomization worked correctly (for each control variable). Can this be added? Also, do the authors have any more demographics available to provide additional evidence? | We only used one randomization procedure. We randomized within each wave of the study without restrictions, and therefore more participants were allocated to the intervention group than the control group.  In a small sample like this, we should rather have forced the allocation to be the same in the two groups by using block randomization. We have elaborated on this weakness in the discussion.  There has been some debate whether one should make statistical test to verify that the baseline data do not differ with respect to the control variables (see for instance de Boer et al. 2015).  We have chosen to follow the consort 2010 recommendation. They state that the significant tests of baseline data are superfluous and can mislead readers (Moher et al. 2010). In an RCT we already know that differences in baseline data are a result of chance rather than bias (Moher et al. 2010).  de Boer, M. R., Waterlander, W. E., Kuijper, L. D. J., Steenhuis, I. H. M., & Twisk, J. W. R. (2015). Testing for baseline differences in randomized controlled trials: An unhealthy research behavior that is hard to eradicate. International Journal of Behavioral Nutrition and Physical Activity, 12(1), 1–8. https://doi.org/10.1186/s12966-015-0162-z  Moher, D., Hopewell, S., Schulz, K. F., Montori, V., Gøtzsche, P. C., Devereaux, P. J., … Altman, D. G. (2010). CONSORT 2010 explanation and elaboration: updated guidelines for reporting parallel group randomised trials. BMJ (Clinical Research Ed.), 340. https://doi.org/10.1136/bmj.c869 | P17, L  We used simple randomization within each of the four waves of the study. As a result of this procedure, 146 participants were allocated to the intervention group and 121 participants were allocated to the control group. In a small sample like this, one could argue that we should have ensured equal allocation to the two groups by using block randomization. However, even with the simple randomization procedure we used, the difference in the two groups are the result of chance rather than bias. |
| The authors state that “incentives can affect the response rate distribution indirectly by affecting the demographic composition of the study sample or by directly affecting the attitude of the respondents.” While the authors test for differences in survey responses across treatment and control group respondents, they don’t offer an extensive look at the demographics of responders and non-responders in each group (apart from gender and age). Do the authors have any more demographics they could use to investigate to what extent the intervention could have affected the demographic composition of the study sample. | We do only have data for gender and age for the study population. We have now specific mentioned gender and age in the introduction. | P4, L63-65  Incentives can affect the response distribution indirectly by affecting the composition of demographic variables, such as gender and age of the study sample, or directly by affecting the attitude of the respondents [8]. |
| RESULTS  Were those that responded representative of the target population overall? (See notes on demographics above). | We do only have information about the demographic variables gender and age. Age and gender were insignificant in the analysis. However, we have included an additional analysis adjusting for group characteristic of the children and youths, see last point. |  |
| It would be helpful to provide not only the response rate and gender percentages but also the raw numbers of individuals (stratified by gender) that participated in the study and responded to the incentives. This way, the reader can clearly see that while these incentives may work better on men, they still do increase the response rate for women as well (in addition to the RR results). For example, these are back-of-the envelope calculations:  Treatment Group  96 women  47 Men  Treated Responders  69 women (72% of women in the treatment group responded)  39 men  Control Group  82 women  37 men  Control Responders  41 women (50% of women in the control group responded)  10 men | We have merged tables 1 and 5 together. We have included the number of individuals stratified by gender in the table. |  |
| Overall, the paper would be stronger if the authors make a better case for why these findings can truly be generalized elsewhere. How similar is the participant population to populations elsewhere? And across the participants (lonely children, foster children, and vulnerable children), are there differences in response rates? As “lonely” is not defined, an analysis of foster and vulnerable children alone could be a useful addition. | The population consists of children and youth from families with severe social problems. All participants shared the same problem of having limited social relations and they were consequently lonely or at risk of being lonely. This has been described in several parts of the manuscripts. We hope this is much clearer now.  We have included a more detailed description of the groups that characterizes the children and youths. The groups are also included in table 1.  We have made an additional analysis where we have adjusted the rate ratio for the group that characterizes the children and youths.  This did not change the rate ratio and the group variable was insignificant in the analysis.  We have updated the different sections according to this analysis. | P11, L219-223  The original study was organised into twelve projects within the ten municipalities. The projects all worked at creating networks for the participating children and youths. The population consisted of 67 children in foster care or former foster care youths, 77 children and youths from vulnerable families and 60 participants characterized as lonely children and youths, Table 1. A total of 58 participants came from projects that included participants from all target groups, Table 1.  P10, L195-196  In a final robustness test, we calculated the rate ratio where we adjusted for the group that characterized the children and youths.  P15, L273-275  In a final analysis, we adjusted the primary analysis for the group variable that characterized the children and youths. This did not change the rate ratio (RR 1.73 CI 1.38-2.17). The group variable was insignificant in the analysis.  P17, L316-318  Furthermore, in the analysis where we adjusted the primary analysis for the group variable that characterized the children and youths, we did not see any change in the rate ratio. |
| While revising your submission, please upload your figure files to the Preflight Analysis and Conversion Engine (PACE) digital diagnostic tool, https://pacev2.apexcovantage.com/. PACE helps ensure that figures meet PLOS requirements. To use PACE, you must first register as a user. Registration is free. Then, login and navigate to the UPLOAD tab, where you will find detailed instructions on how to use the tool. If you encounter any issues or have any questions when using PACE, please email us at figures@plos.org. Please note that Supporting Information files do not need this step |  |  |
